# Surface Response Analysis for the Optimization of Mechanical and Thermal Properties of Polypropylene Composite Drawn Fibers with Talc and Carbon Nanotubes

**DOI:** 10.3390/polym14071329

**Published:** 2022-03-25

**Authors:** Konstantinos Leontiadis, Costas Tsioptsias, Stavros Messaritakis, Aikaterini Terzaki, Panagiotis Xidas, Kyriakos Mystikos, Evangelos Tzimpilis, Ioannis Tsivintzelis

**Affiliations:** 1 Department of Chemical Engineering, Aristotle University of Thessaloniki, University Campus, GR-54124 Thessaloniki, Central Macedonia, Greece; leontiad@cheng.auth.gr (K.L.); tzimpi@auth.gr (E.T.); 2 Plastika Kritis S.A., R Street, Industrial Area of Heraklion, GR-71408 Heraklion, Crete, Greece; messaritakis@plastikakritis.com (S.M.); terzaki@plastikakritis.com (A.T.); 3 Thrace Nonwovens & Geosynthetics S.A., Magiko, GR-67100 Xanthi, East Macedonia and Thrace, Greece; pxidas@thraceplastics.gr (P.X.); kmystikos@thraceplastics.gr (K.M.)

**Keywords:** drawn polymer fibers, polymer composites, polypropylene, talc, carbon nanotubes

## Abstract

A large portion of the produced Polypropylene (PP) is used in the form of fibers. In this industrially oriented study, the development of composite PP drawn fibers was investigated. Two types of fillers were used (ultra-fine talc and single-wall carbon nanotubes). Optimization of the thermal and mechanical properties of the produced composite drawn fibers was performed, based on the Box-Behnken design of experiments method (surface response analysis). The effect of additives, other than the filler, but typical in industrial applications, such as an antioxidant and a common compatibilizer, was investigated. The drawing ratio, the filler, and the compatibilizer or the antioxidant content were selected as design variables, whereas the tensile strength and the onset decomposition temperature were set as response variables. Fibers with very high tensile strength (up to 806 MPa) were obtained. The results revealed that the maximization of both the tensile strength and the thermal stability was not feasible for composites with talc due to multiple interactions among the used additives (antioxidant, compatibilizer, and filler). Additionally, it was found that the addition of talc in the studied particle size improved the mechanical strength of fibers only if low drawing ratios were used. On the other hand, the optimization targeting maximization of both tensile strength and thermal stability was feasible in the case of SWCNT composite fibers. It was found that the addition of carbon nanotubes improved the tensile strength; however, such improvement was rather small compared with the tremendous increase of tensile strength due to drawing.

## 1. Introduction

Polypropylene (PP) and polyethylene are the most popular thermoplastics. PP’s chemical inertia, mechanical strength, low electric and thermal conductivity, high melting point, and easy processing render it as a very important polymeric material for a wide range of applications. In most applications, PP is used either in the form of films/sheets or in the form of fibers. Films and sheets are mainly used as packaging materials, while fibers are mainly used in textiles. Other applications include automotive and medical products industry. The COVID-19 pandemic outbreak further revealed the industrial significance of PP-based materials, where the increased need for disposable PP non-woven fabrics for gowns, face masks, and shoe covers has appeared [1]. Consequently, the improvement of both mechanical and thermal properties of PP based materials is a research area of continuous interest.

Regarding PP fibers, it is widely known that a significant increase of mechanical strength can be achieved by drawing, under appropriate conditions (e.g., appropriate drawing ratio and temperature) due to the alignment of macromolecular chains in the drawing axis direction [2,3,4,5,6,7,8,9,10,11,12]. For example, the use of a drawing ratio equal to 7 can substantially increase the PP fiber’s tensile strength from around 30–40 MPa to around 400–500 MPa [11,12]. Besides macromolecules, it has been observed that low molecular weight needle-like fillers also align during drawing, which results in a more efficient and uniform transfer of mechanical stresses from the polymer to the filler [5,6,13,14].

Fillers are widely used in order to alter various properties of PP. Depending on the filler type, enhancement in mechanical [15,16,17,18], thermal [15,16,17,19], and electrical [19,20,21] properties can be achieved. For example, the addition of only 0.5% wt. of modified montmorillonite nanoparticles in the PP matrix increased the tensile strength of drawn fibers from 532 MPa (neat PP) to 690 MPa (composite material) [15]. Additionally, the addition of modified montmorillonite in the PP matrix increased the onset decomposition temperature by 76 °C [17]. Skrifvars et al. reported an electrical conductivity value of 2.8 S cm^−1^ for PP/multi-wall carbon nanotube (MWNT) composite-drawn fibers, compared with the 10^−10^ S cm^−1^ of neat PP. The current trends in PP composite drawn fibers have been recently reviewed [22].

Polypropylene, as a polyolefin, has a hydrophobic and non-polar nature, while minerals, commonly used as inorganic fillers, are hydrophilic and polar. Those characteristics hinder the homogenous dispersion of the filler in the polymer matrix, resulting in filler aggregates that not only do not improve, but, on the contrary, may deteriorate the final properties of the composite material. A better dispersion can be achieved by improving the interaction between the filler and the polymer. A common practice for polypropylene composites is the use of compatibilizers, with PP grafted with maleic anhydrate (PP-g-MA) being the most popular one [6,17,23,24,25,26,27]. Functionalization/modification of fillers’ surface is also common within the numerous fillers. Montmorillonite (MMT) has been broadly modified with quaternary ammonium salts [17,23,24]. Wollastonite has been treated with acids in order to be used in polypropylene composites [28,29]. Tambe et al. used functionalized multi-wall carbon nanotubes [6], while McIntosh et al. used functionalized, with fluorine on their surface, multi-wall carbon nanotubes, resulting in covalent bonding between the nanotubes and the PP matrix [30].

In this study, two fillers were used, namely, carbon nanotubes (CNT) and talc. CNTs are usually used at low content (about 0.5–1% wt.), while talc is used at high content (up to 50% wt.) [31]. There are plenty of studies using CNTs as fillers for drawn polypropylene composite fibers [6,16,18,19,21,30,32,33,34,35,36,37], whereas there is a paucity of research studies for polypropylene composite drawn fibers using talc as a filler [38,39]. Values of tensile strength in the range 300–1000 MPa have been reported in PP-drawn fibers with carbon nanotubes, e.g., for PP-amine functionalized carbon nanotubes [6] or for non-functionalized carbon nanotubes [36]. Fluorinated carbon nanotubes at a content of 10% induced a 150% increase of the maximum stress of PP fibers (from 30 to 77 MPa) [30]. However, optimization based on response surface analysis has not been performed in any of these studies.

Another issue that is usually ignored in studies dealing with PP composite fibers is the synergistic or competitive effects of various additives, e.g., antioxidants, coloring, and UV-protection agents, which are usually incorporated in the polymer matrix of industrial products. In this direction, Tsioptsias et al. revealed that the use of PP-g-MA as a compatibilizer in PP composites results in a better dispersion of a phenolic type antioxidant, which in turn results in a more pronounced thermal protection during processing and, indirectly, in increased mechanical properties of the final drawn polymer fibers [11,12]. However, the increased compatibilizer–antioxidant (additive) interactions may act competitively to the compatibilizer–filler interactions. Thus, the dependence of a desired property, e.g., the tensile strength, on the content of the compatibilizer or the antioxidant is expected to be non-linear. Consequently, response surface analysis is a very useful tool in order to study non-linear and multi variable effects on the desired properties (response variables) [40,41]. Recently, surface response methodology was used to optimize the properties of PP-wollastonite composite drawn fibers [12]. The optimum values of the response properties that were predicted by the developed model were in very good agreement with subsequent verification experiments [12].

This study is the third part of a series of papers [11,12] related to the development and optimization of the properties of PP-composite drawn fibers that also contain some typical industrial additives. Firstly, preliminary experiments were carried out using a fixed drawing ratio of 7 and fixed compatibilizer and antioxidant content [11]. These preliminary experiments showed that some fillers (microtalc and attapulgite) are not promising for the PP-drawn fibers applications, while other fillers (wollastonite, ultra-fine talc and carbon nanotubes) might contribute to the development of PP-based materials with enhanced properties and deserve further investigation. Additionally, various interactions and competitive/synergistic effects between the various additives (filler, antioxidant, compatibilizer) were recognized. As a continuation of that work, the development of PP-wollastonite drawn fibers was investigated by surface response analysis over a wide range of compatibilizer content and drawing ratios in the range of 5 to 9 [12]. In this work, PP composite drawn fibers with the other two promising fillers (ultra-fine talc and carbon nanotubes) were also investigated and the properties were optimized based on surface response analysis. New variables were included in the design of experiments (antioxidant content), while other variables (e.g., compatibilizer content and drawing ratio) were investigated in new ranges in order to provide further insights for the competitive and synergistic effects among the various additives under the influence of process parameters, e.g., to find the upper limit for the drawing ratio in needle-like fillers, such as the SWCNTs.

## 2. Experimental

### 2.1. Materials

In all cases, isotactic polypropylene was mixed with masterbatches containing the used additives, i.e., talc, SWCNTs, compatibilizer, and antioxidant. The most important characteristics of the used materials are shown in Table 1.

### 2.2. Experimental Apparatus and Procedure

The experimental apparatus and procedure have been thoroughly described in previous studies [11,12]. Briefly, the mixing of PP with the additive containing masterbatches was performed in a twin screw extruder (HAAKE Rheodrive 5001) with four heating zones that were set at 190, 210, 215, and 220 °C from feed to die, using 25 rpm as the rotating screw speed. The produced filament was pelletized and then fed into a single-screw extruder (Xcalibur, Noztec, Shoreham-by-Sea, United Kingdom) with three heating zones that were set at 215, 225, and 210 °C, from feed to die, and using a motor speed equal to 15 rpm. Then, the produced composite filament was collected, marked every 5 cm lengthwise, and drawn at the desired drawing ratio. Solid-state drawing was performed at 140 °C, which is below the melting point of the composites that in all cases ranged between 161 and 171 °C. After drawing, parts of the fibers that had the expected distance between two marks (as expected by the chosen drawing ratio) were selected and characterized.

### 2.3. Characterization

Differential scanning calorimetry (DSC) measurements, using a Shimadzu DSC-50 apparatus, and thermogravimetric analysis (TGA), using a Shimadzu TGA-50 equipment, were performed for the determination of melting temperature, heat of fusion and onset decomposition temperature, respectively. DSC scans were performed using a heating rate of 10 °C min^−1^ from ambient temperature up to 230 °C and under nitrogen atmosphere (flow rate equal to 20 mL min^−1^). DSC samples were weighed using a Sartorius B120s scale (with accuracy of 0.0001 g). TGA measurements were conducted with a heating rate of 20 °C min^−1^, under air atmosphere, from ambient temperature up to 450 °C.

Tensile strength measurements were performed using a Hans Schmidt & Co GmbH Universal Testing Machine ZPM (Waldkraiburg, Germany), equipped with a Pacific PA6110 loadcell (head speed 100 mm min^−1^). The presented values for tensile strength are the averages of 8–12 measurements using random pieces of the drawn fibers.

## 3. Design of Experiments

Optimization of fiber properties was performed using a surface response methodology through design of experiments (DoE) selected using the Box-Behnken approach. The Minitab^®^ 20.4 software was used.

Two sets of DoE were performed, i.e., one for each filler. Considering the composites with talc, the filler content (0–4% wt.), the content of the compatibilizer masterbatch (0–7.5% wt.), and the drawing ratio (7 to 9) were chosen as design variables. In the case of composites with SWCNTs, the filler content (0–1% wt.), the content of the antioxidant masterbatch (0–8% wt.), and the drawing ratio (7 to 21) were selected as design variables.

In both cases, the tensile strength (*TS*), the onset decomposition temperature *(T_dec_*), defined as the temperature at which the remaining mass is 97% of the initial mass (i.e., 3% wt. mass loss), the melting temperature (*T_m_*), and the melting enthalpy (Δ*H*) were chosen as response variables. Representative stress-strain, DSC, and TGA curves can be found in the Supplementary Materials file (Figures S1–S3, respectively).

The values of the design variables, for each experiment, are presented in Table 2 and Table 3 for DoE1 (composites with talc) and DoE2 (composites with carbon nanotubes), respectively. All PP/talc composites contained 4% wt. of the antioxidant masterbatch, which was also used in a previous study [11].

The concept for selecting the aforementioned ranges for each design variable is presented in the next lines. For the case of talc, the selected filler content range is typical in the development of various nanocomposites, in contrast to microcomposites, which usually require much higher filler contents, e.g., up to 20% wt. Additionally, the range of the compatibilizer content was selected based on our previous findings for PP-wollastonite fibers [11,12]. In more detail, it was observed that compatibilizer contents up to 15% wt. did not contribute to the enhancement of final properties, in contrast with the relatively lower contents of 1.5% wt. [11,12]. Thus, in the current study, a shorter range was chosen to be studied (0–7.5% wt.). Additionally, in our previous work [11,12], we found that drawing ratios up to 9 do not cause deterioration of mechanical properties due to overstretching and, by taking into account that the talc composites will be susceptible to overstretching due to high contents of masterbatches with low molecular weights (compatibilizer and antioxidant), 9 was selected as the limit of the drawing ratio.

On the contrary, for the PP-SWCNT composites, no compatibilizer was used, since, in contrast with minerals, the filler is not hydrophilic. In addition, due to the needle-like morphology of SWCNTs (ability to align along the drawing direction), higher drawing ratios could be beneficial. Consequently, a wide range of the drawing ratio was selected to be investigated. Additionally, in a previous study, a fixed content of 4% wt. for the antioxidant masterbatch was used and was found to have a beneficial effect. Thus, in order to study its effect at higher contents, a wider range (0–8% wt.) was selected. Finally, the range for the SWCNT content was again based on typical values used in nanocomposites, but also by taking into account the much higher cost of SWCNTs. Thus, lower contents (compared with those of talc) were chosen for the case of SWCNTs (0–1% wt.).

## 4. Results and Discussion

### 4.1. Composites with Talc (DoE 1)

In Table 4, the results for the 15 samples of DoE1 (DoE for talc composites) are presented. More precisely, the four response variables, namely, tensile strength (*TS*), decomposition temperature (*T_dec_*), melting enthalpy (Δ*H*), and melting temperature (*T_m_*) are shown, along with the three design variables, i.e., talc content, compatibilizer masterbatch content, and drawing ratio (*λ*). Some representative plots are presented in Figures S1–S3 of the Supplementary Materials file. As discussed in our previous work [12], 8–12 tensile tests were performed for each sample; however, this is not feasible for TGA and DSC measurements. The uncertainty for Δ*H*, as well as for the decomposition and melting temperatures are very low (estimated by the instrument sensitivity), while the uncertainty for *TS* is high since is it calculated as the standard deviation of 8–12 measurements. In addition, the last three experiments (number 13, 14, and 15) in each design of experiments are three independent repetitions of the same sample. The deviation of the values of the response variables among these three repetitions is taken into account by the model (fitting procedure). Having that in mind and in order to avoid any misleading conclusions, the very low uncertainties for *T_dec_*, *T_m_,* and Δ*H* are not presented, and only the errors for *TS* are presented in the Tables with DoE results.

The values presented in Table 4 were used for the surface response analysis. It is worth mentioning that the model presented moderate *R-sq* values (0.68–0.76) and *p*-values much higher than 0.05 for *T_de_*_c_, Δ*H*, and *T_m_*, while for tensile strength (*TS*) it presented *R-sq* equal to 0.897 and a *p*-value equal to 0.049 (see Table S1 of the Supplementary Materials file). A high *p*-value indicates a lack of statistical significance. For this and other reasons, which are explained next, *T_m_* and Δ*H* were not taken into account in the optimization procedure. Although the fitting for the *T_dec_* cannot be considered satisfactory (statistically significant), *T_dec_* fluctuates in a very short range, as can be seen in Table 4, and thus any error in the prediction from the model is expected to be low. Thus, *T_dec_* was not fully neglected during optimization.

#### 4.1.1. Single Design Variable Effect on Response Variables for Composites with Talc

**Figure 1:**
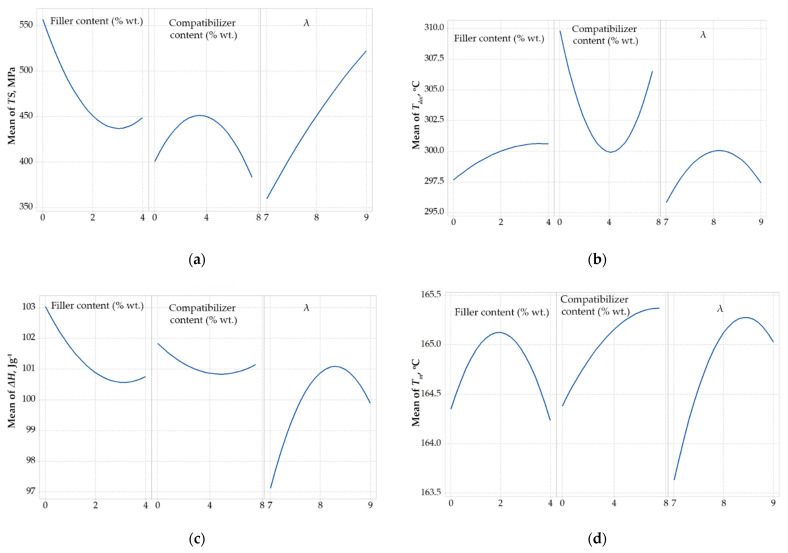
Correlation of design variables with (**a**) Tensile strength, *TS*, (**b**) Onset decomposition temperature, *T_dec_*, (**c**) Heat of fusion, Δ*H*, and (**d**) melting point, *T_m_*, regarding composites with talc.

In Figure 1 the main effects plots for the response variables are shown, considering the composites with talc. All response variables are non-linearly related to the design variables.

As shown in Figure 1a, except the almost linear relation between the drawing ratio and the tensile strength, all other responses showed a non-linear behavior, which is an indication of multiple (e.g., antagonistic) effects. For example, as shown in the middle plot of Figure 1a, the addition of compatibilizer (PP-g-MA) up to 4% wt. improved the tensile strength, while further increment of its content resulted in a weakening of mechanical strength. This can be explained by the multiple effect of the compatibilizer. Firstly, as shown by Tsioptsias et al., it facilitates the dispersion of both the filler and the antioxidant, tending to increase the tensile strength, but at the same time, its lower molecular weight results in limited stretching potential, deteriorating the mechanical properties of the drawn fibers [11].

Additionally, as shown by the left plots of Figure 1a,b, the addition of talc up to about 3% wt. deteriorated the tensile strength, while, at the same time, increased the onset decomposition temperature. On the other hand, as shown by the right plots of Figure 1a,b, the drawing ratio had a positive effect on tensile strength in the studied range, while its effect on decomposition temperature showed a maximum. However, this influence on *T_dec_* is minor, since the values of *T_dec_* only ranged between 296 and 300 °C.

Furthermore, as presented in Figure 1c,d, Δ*H* and *T_m_* were affected only to a low extent from the alteration of design variables (in the studied range). In addition, for industrial applications, the tensile strength and the thermal stability are of primary interest. Consequently, Δ*H* and *T_m_* were not further considered and the subsequent optimization approaches were focused only on the tensile strength and the onset decomposition temperature.

#### 4.1.2. Combined Effect of Design Variables on Response Variables for Composites with Talc

**Figure 2:**
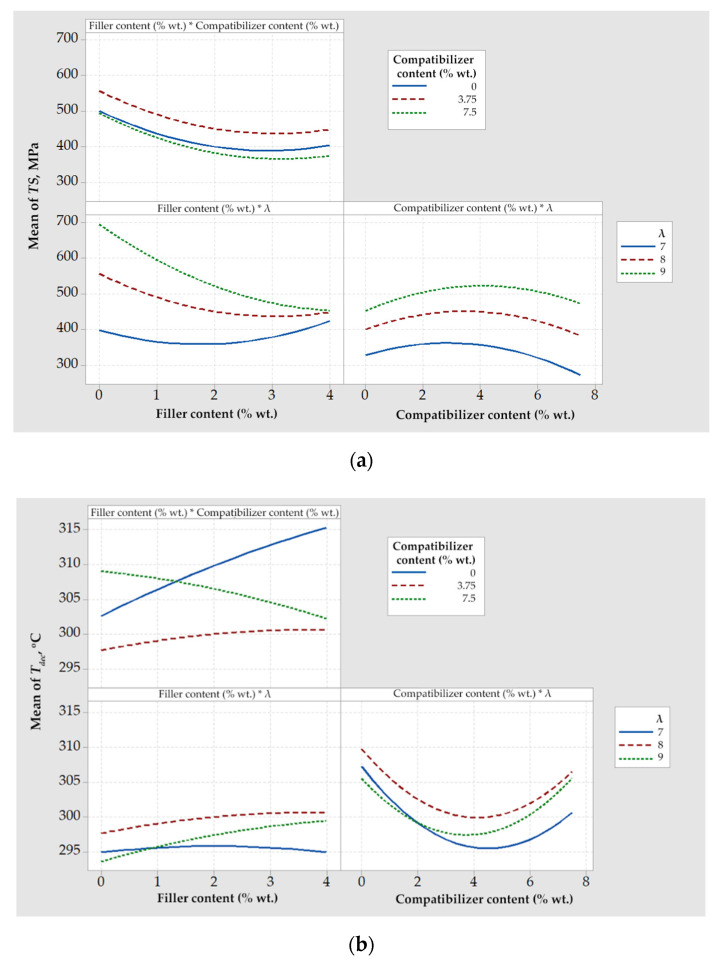
Interaction plots for (**a**) tensile strength, *TS*, and (**b**) onset decomposition temperature, *T_dec_*, regarding composites with talc.

Interaction plots are presented in Figure 2 for the tensile strength (Figure 2a) and the onset decomposition temperature (Figure 2b). Parallel lines indicate that no interaction occurred, while nonparallel lines indicate the opposite. In terms of tensile strength, the strongest interaction was observed among talc content and drawing ratio (as shown in by the left lower plot of Figure 2a). In this graph it can be seen that for a drawing ratio equal to 7, filler addition over 3% wt. reinforced the composite fibers, while for drawing ratios equal to 8 or 9, the addition of talc deteriorated their mechanical properties. Most likely, the presence of talc interferes with the increased chain alignment; thus, at high drawing ratios, any positive effect on *TS* by the presence of talc itself cannot compensate its negative effect on the chain alignment.

A strong interaction could also be observed for the talc and the compatibilizer content on the onset decomposition temperature (left upper plot of Figure 2b). In the absence of compatibilizer, or for compatibilizer content equal to 3.75% wt., an increase in the talc content increased the decomposition temperature, as expected. However, for a high compatibilizer content (e.g., 7.5% wt.), this effect was reversed. This observation indicated the existence of multiple effects, e.g., as determined in our previous study [11], increased compatibilizer-antioxidant and filler-antioxidant interactions that may act competitively to the filler-compatibilizer interactions. Based on such multiple and competitive effects, the necessity of optimization becomes apparent. This is discussed in the next section.

#### 4.1.3. Optimization Runs for Composites with Talc

**Figure 3:**
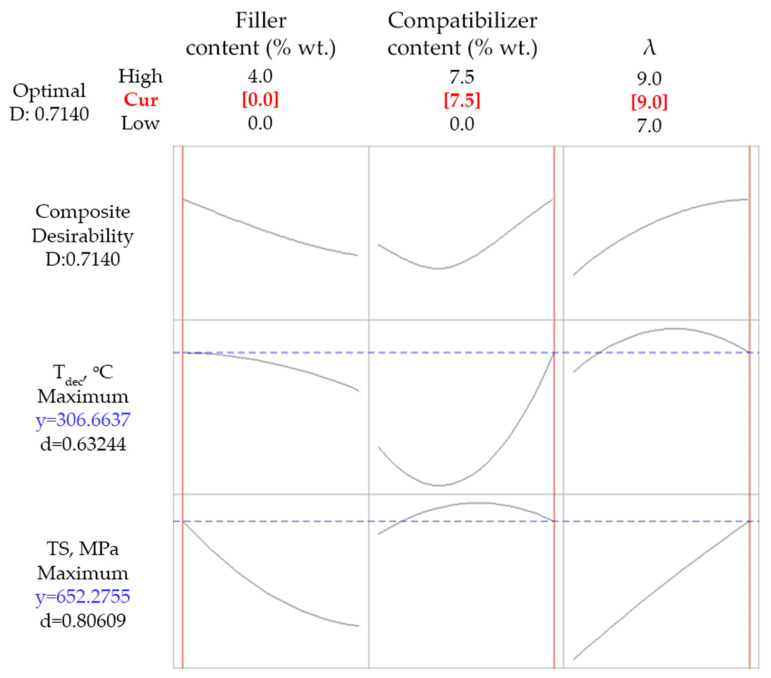
Optimization results targeting simultaneous maximization of *TS* and *T_dec_* for composites with talc.

Several optimization scenarios were tested. Firstly, a simultaneous maximization of *TS* and *T_dec_* was investigated and the results are shown in Figure 3.

As it can be concluded by the rather low value of the composite desirability (0.714), the simultaneous maximization of tensile strength and thermal stability cannot be fulfilled. The achieved values of the response variables were 652 MPa for *TS* and 307 °C for *T_dec_*, which were obtained for 7.5% wt. compatibilizer content (maximum of the studied range), 0% wt. talc content (minimum of the studied range), and a drawing ratio equal to 9 (maximum of the studied range). The unfeasibility of maximizing both the tensile strength and the onset decomposition temperature was also observed in PP-wollastonite drawn fibers using the same antioxidant and compatibilizer and it was attributed to the dual effect of the compatibilizer [12]. Having in mind its lower, relatively to the used PP matrix, molecular weight (as indicated by *MFI* in Table 1) and its lower thermal stability [11], the deterioration of both the thermal stability and the tensile strength of the composite material is expected upon increasing its content. On the other hand, upon adding compatibilizer the dispersion of the filler and the antioxidant are favored, as reported in our previous work [11], tending to indirectly improve both thermal and mechanical properties. The net effect of such phenomena is the simultaneous existence of the maximum in the *TS* curve shown in Figure 1a and the minimum of the *T_dec_* curve shown Figure 1c (plots in the middle in both cases). Consequently, there is no way of maximizing both of them.

**Figure 4:**
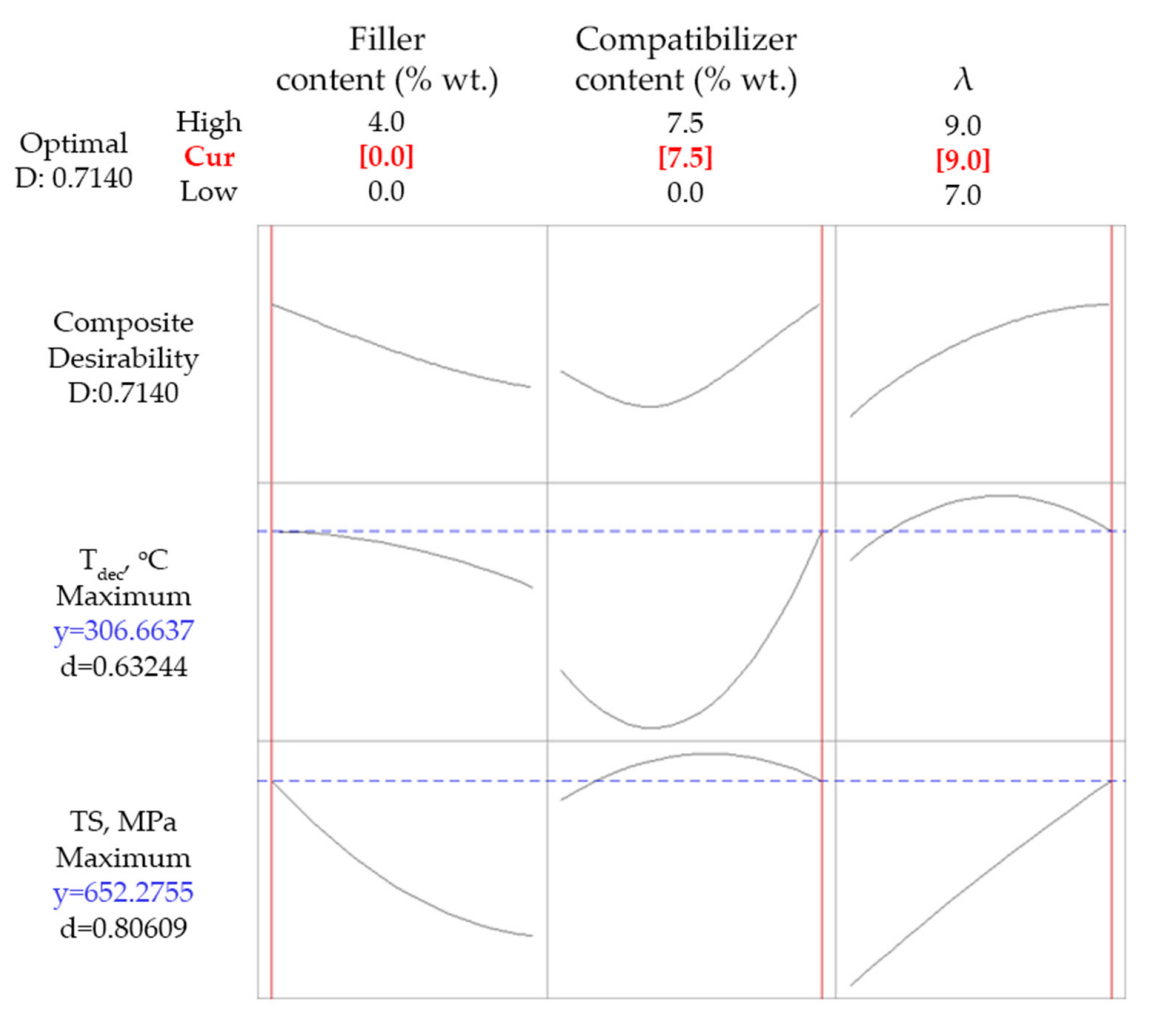
Optimization results targeting maximum *TS* and *T_dec_* at least equal to 300 °C for composites with talc.

Since the simultaneous maximization of *TS* and *T_dec_* cannot be fulfilled, an optimization based on a compromise of *T_dec_* at least equal to 300 °C (which represents a fair improvement of thermal stability) and maximization of *TS* was tested. As is shown in Figure 4, the criterion for *T_dec_* was fully met, while *TS* was predicted equal to 682 MPa, showing a desirability value of 0.87. Such values of the response properties were obtained for 6.1% wt. compatibilizer content, 0% wt. talc content (minimum of the studied range), and a drawing ratio equal to 9 (maximum of the studied range).

**Figure 5:**
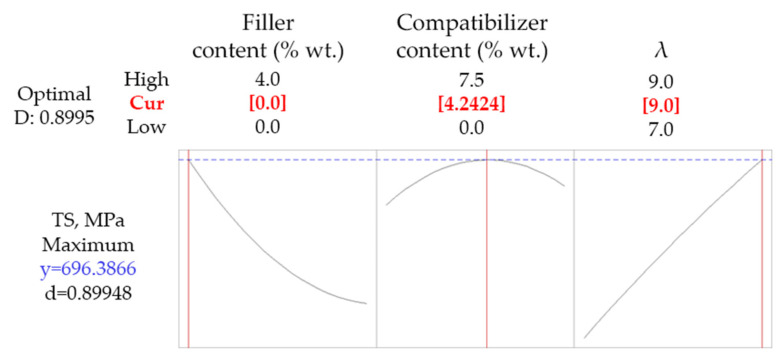
Optimization results targeting maximum *TS* for composites with talc.

Finally, since tensile strength is more important than thermal stability in most applications, a final optimization run was performed using the maximization of *TS* as the only criterion. Results are presented in Figure 5. *TS* of 696 MPa was predicted for 0% wt. talc content (minimum of the studied range), about 4.2% wt. compatibilizer content, and a drawing ratio equal to 9 (maximum of the studied range). In this case, the desirability showed a rather high value equal to 0.9.

From the above results, it becomes apparent that all three optimization approaches were in agreement regarding the optimum values for the drawing ratio and the talc content. If no maximization of *T_dec_* is required, then the optimum value for the compatibilizer content ranges between 4.2 and 6.1% wt. In this case, the improvement of properties arose from the more effective dispersion of the antioxidant upon the existence of the compatibilizer.

**Figure 6:**
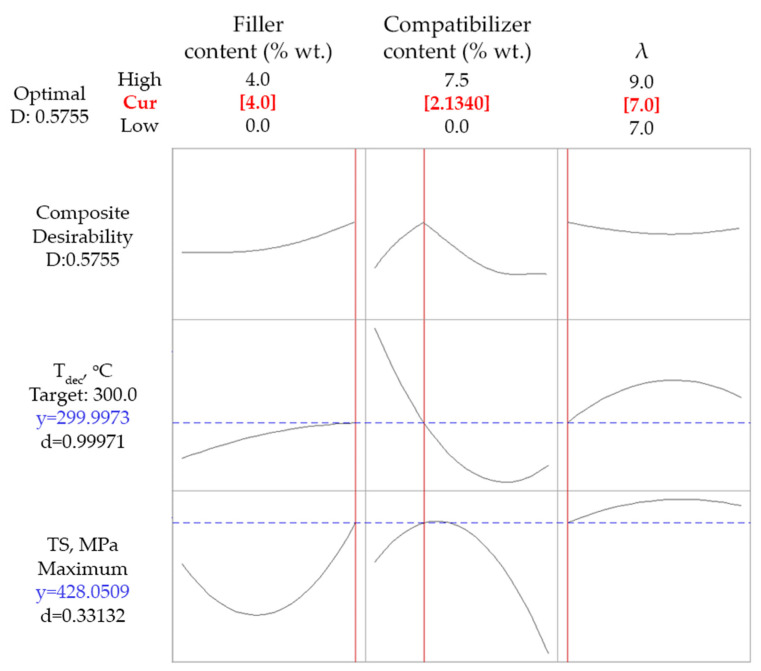
Constrained optimization results for composites with talc, imposing a drawing ratio of 7 and targeting maximization of *TS* and *T_dec_* at least equal to 300 °C.

The absence of talc from the above optimum compositions was expected, since, as discussed in the previous section, talc, in the used particle size, most likely interferes with the increased chain alignment and, thus, at high drawing ratios, any positive effect on *TS* by the presence of talc itself cannot compensate for its negative effect on chain alignment. However, as shown in Figure 2a (left lower plot), such a negative effect on chain alignment is not the dominating phenomenon for the lower implemented drawing ratio of 7. In this particular case, addition of talc over 3% wt. reinforced the composite fibers. Consequently, and since a drawing ratio of 7 is usually applied in industrial practice, a final optimization run was executed, using a drawing ratio of 7 and targeting maximization of *TS* and a decomposition temperature at least equal to 300 °C. The results are presented in Figure 6. For a composition of 4% wt. talc (maximum of the investigated range) and 2.1% wt. compatibilizer, the *T_dec_* criterion was fully met, while the model predicted a tensile strength, which is a fair increment compared with the neat PP drawn fibers.

#### 4.1.4. Verification—Comparison with Literature

In order to compare model predictions with experiments, the repeatability of measurements should be considered. This was accounted for through the applied Box-Behnken approach, since three out of the total fifteen experiments were independent repetitions (experiments number 13, 14, and 15 of Table 4) of the same experiment. As shown by the obtained values, in all three cases there was an excellent repeatability considering the melting temperature (*T_m_*) and the Heat of fusion (Δ*H*), while the standard deviation of the three values for the onset decomposition temperature was 1.9 °C and for the tensile strength 19.4 MPa.

Regarding the results of Figure 6, using a drawing ratio of 7.4% wt. of the antioxidant masterbatch (0.82% wt. of the active ingredient), 4% wt. talc, and 2.1% wt. of the compatibilizer masterbatch, the model predicted a *TS* equal to 428 MPa and a *T_dec_* equal to 300 °C. These values are in fair agreement with preliminary experimental results for a sample with similar composition (the only difference is the slightly lower compatibilizer content, which was equal to 1.5% wt.) that are presented in our previous study [11], i.e., *TS* equal to 394 ± 46 and *T_dec_* equal to 297 °C.

### 4.2. Composites with SWCNTs (DoE 2)

In Table 5, results for the fifteen fiber samples of DoE2 (composites with SWCNTs) are presented. The obtained values of the four response properties, i.e., tensile strength (*TS*), onset decomposition temperature (*T_dec_*), melting enthalpy (Δ*H*), and melting temperature (*T_m_*) are presented, along with the values of the three design variables, namely the SWCNT and antioxidant content and the drawing ratio (*λ*).

As for the case of talc, the values presented in Table 5 were used for the surface response analysis (see Table S2 in Supplementary Materials file for *R-sq* and *p*-values). In contrast to the case of PP-talc, in the case of PP-SWCNT composites the fitting for all four response variables was fairly good (*R-sq* values higher than 0.88 and *p* values lower than 0.064 in all cases). We speculate that this arose from the fact that in the PP-SWCNT composites there was no compatibilizer and, thus, a large portion of the multiple effects, caused by its addition, vanished, resulting in less complex behavior. However, only *TS* and *T_dec_* were considered for the optimization procedure as explained in the next sections.

#### 4.2.1. Single Design Variable Effect on Response Variables for Composites with SWCNTs

**Figure 7:**
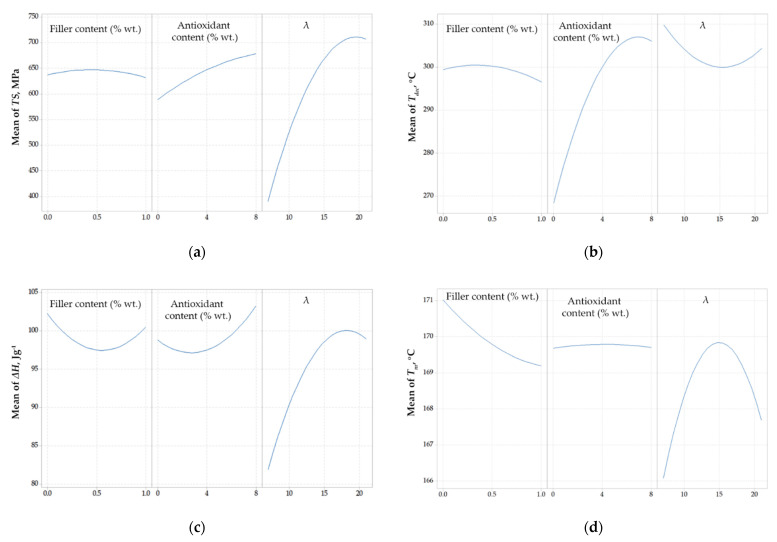
Main effect plots for (**a**) Tensile strength, *TS*, (**b**) Onset decomposition temperature, *T_dec_*, (**c**) Heat of fusion, Δ*H*, and (**d**) Melting point, *T_m_*, regarding composites with SWCNTs.

In Figure 7, the main effect plots for the response variables are presented. It is shown that tensile strength was mostly affected by the applied drawing ratio, while the effects of the filler and the antioxidant were much smaller (Figure 7a). A local maximum of tensile strength was observed at about 0.5% wt. CNT content, which is in agreement with the results of Dondero and Gorga, who used multi-wall carbon nanotubes (MWNT) as fillers for preparing PP composites [16]. Furthermore, in that literature study, PP/MWNT composites containing 0.5% MWNT exhibited tensile strength equal to 620 MPa and 474 MPa for a drawing ratio equal to 12 and 23, respectively [16]. A similar trend was observed in the current study between the drawing ratio and the tensile strength. More precisely, for drawing ratios higher than 18, a deterioration of mechanical strength could be observed due to the overstretching of polymer chains. Furthermore, the positive correlation between the antioxidant content and the tensile strength can be attributed to the fact that the antioxidant protects the polymer from thermal stress/decomposition during the two extrusions, which results in less deterioration of the polymer matrix and, thus, in better observed mechanical properties.

A considerable increase of *T_dec_* could be observed upon increasing the antioxidant content (Figure 7b), while the effect of the filler content and the drawing ratio on the *T_dec_* was not so pronounced. Interestingly, a maximum of *T_dec_* was observed for antioxidant content around 7% wt., which probably is connected to the influence of the antioxidant on the crystallites volume and the crystallinity of the final polymer matrix that is also revealed from Figure 7c. However, the decrease of *T_dec_*, at high antioxidant contents, was very small compared to the increase of *T_dec_* that occurred for antioxidant contents in the range of 0% to 7% wt. Additionally, as shown in the last plot of Figure 7b, the onset decomposition temperature showed a minimum when plotted against the drawing ratio. This behavior should be attributed to two antagonistic phenomena, i.e., increasing the drawing ratio decreases the fibers’ diameter, resulting in faster heat transfer, tending to decrease the observed decomposition temperature, while, at the same time, the higher drawing ratio increases the orientation of the fibers, which leads to the formation of more compact structures, tending to increase the observed decomposition temperature.

As presented in Figure 7c, the drawing ratio, as expected, had the more pronounced effect on the crystallinity of the final polymer matrix (as indicated by the measured heat of fusion). Interestingly, the local maximum of the heat of fusion (see the third plot of Figure 7c) occurred at almost the same drawing ratio, for which the local maximum of tensile strength was observed (compare with the last plot of Figure 7a). The effect of the filler and the antioxidant content on the heat of fusion was not significant. Finally, as shown in Figure 7d, none of the design variables had a significant effect on melting temperature (differences less than 4 °C were observed in all cases).

Since the heat of fusion and the melting temperature were affected only to a low extent from the alteration of design variables (in the studied range) and that only the tensile strength and the thermal stability are of primary interest in most industrial applications, Δ*H* and *T_m_* were not further considered in the optimization approaches that are presented in the next sections.

#### 4.2.2. Combined Effect of Design Variables on Response Variables for Composites with SWCNTs

**Figure 8:**
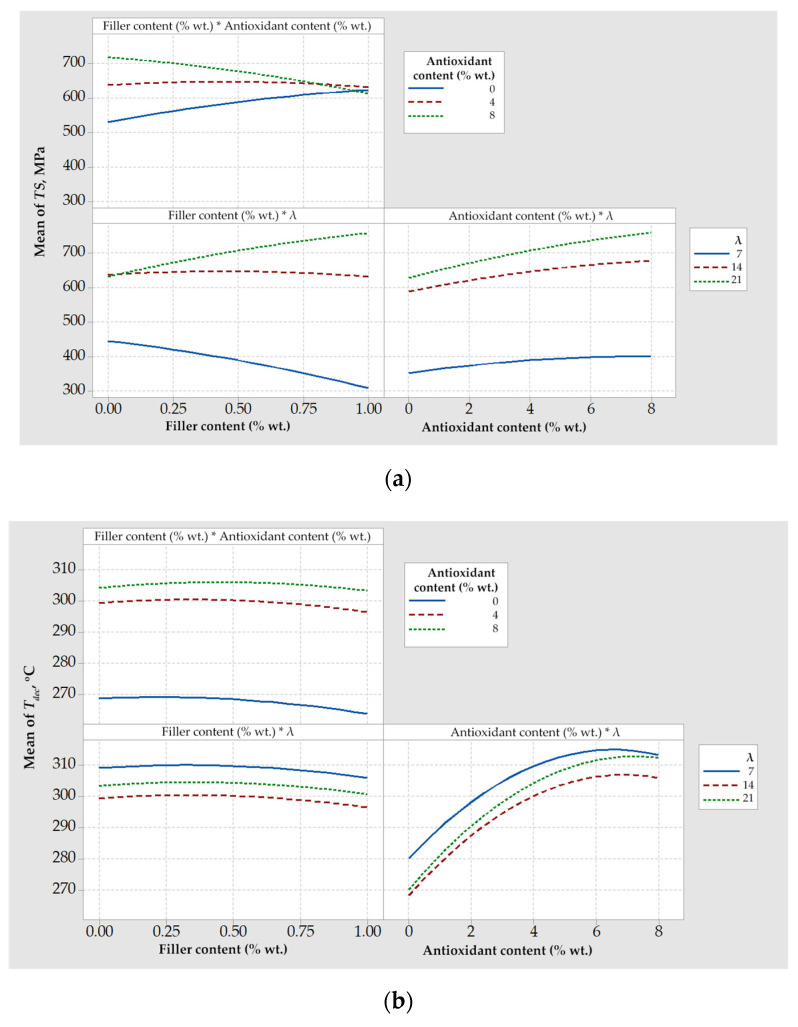
Interaction plots for (**a**) tensile strength, *TS*, and (**b**) onset decomposition temperature, *T_dec_*, regarding composites with SWCNTs.

Interaction plots for the tensile strength, *TS*, and the onset decomposition temperature, *T_dec_*, are presented in Figure 8. As shown by the lower right plot of Figure 8a (Antioxidant content * λ), no significant interaction effect between the drawing ratio and the antioxidant content on tensile strength could be observed. On the contrary, a significant interaction occurred between the drawing ratio and the SWCNT content, as shown by the lower left plot (*Filler content* * *λ*) of Figure 8a. More precisely, for the lowest drawing ratio (7), the addition of SWCNTs caused a decrease of tensile strength, which can be considered as an indication of poor dispersion. At higher values of the drawing ratio (14 and 21), this effect was reversed and for the maximum drawing ratio (21) a clear increase of the tensile strength, by increasing the CNT content, was observed. This can be attributed to the alignment of the filler to the drawing and chain alignment direction, which increases tensile strength as already mentioned in literature [5,6,13,14]. It is evident that the drawing ratio of 7 is not enough to adequate align the SWCNTs, while at higher drawing ratios a better alignment was achieved.

Furthermore, a rather strong interaction effect between the SWCNT and the antioxidant content on tensile strength was revealed by the presence of non-parallel curves in the respective plot (upper left plot, Filler content * Antioxidant content) of Figure 8a. It can be observed that tensile strength was positively affected by the increase of the SWCNT content, in absence of antioxidant, while the opposite behavior was observed upon antioxidant’s presence. This can be attributed to the polar groups of the antioxidant (combination of phenolic and phosphite type) that interact with the filler [11] and, thus, hinder the PP-SWCNT interactions, resulting in poorer dispersion.

Finally, the almost-parallel curves in the plots of Figure 8b reveal that no considerable interaction effect of the design variables occurred on the decomposition temperature.

#### 4.2.3. Optimization Runs for Composites with SWCNTs

**Figure 9:**
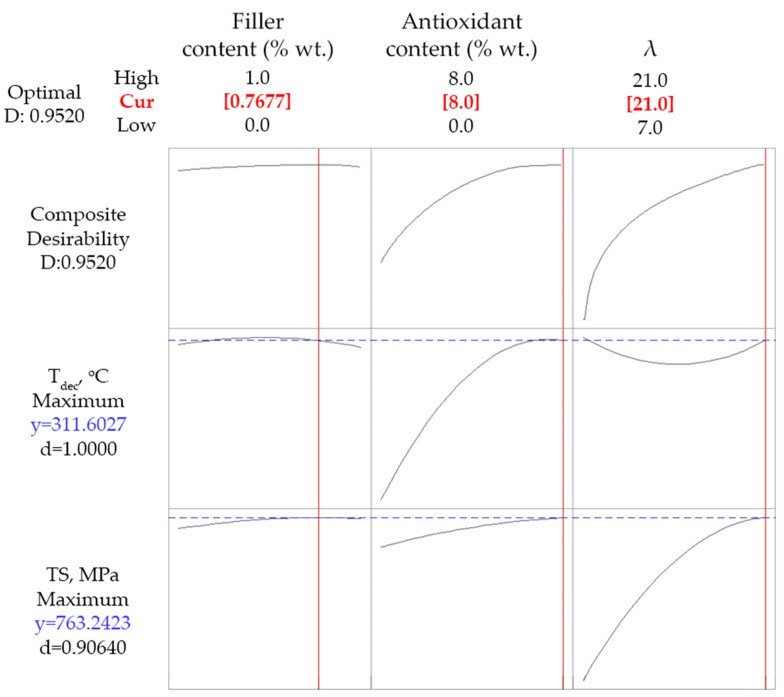
Optimization results targeting maximum *TS* and *T_dec_* for composites with SWCNTs.

The optimization approaches that were used for DoΕ1, which were presented in a previous section for the PP-talc composites, were also tested for the case of PP-SWCNT composites. Firstly, a simultaneous maximization of *TS* and *T_dec_* was targeted and the results are presented in Figure 9. Contrary to the case of talc, in this case high values of partial and composite desirability were obtained. Those desirability values were interpreted to 311 °C and 763 MPa, for *T_dec_* and *TS*, respectively, for a composite with maximum antioxidant (8% wt.), 0.77% wt. CNT content and a maximum drawing ratio (21).

Similar results and composition were achieved for a second optimization run, considering the maximization of *TS* as the only criterion. The relevant results are presented in Figure S4 of the Supplementary Materials file. This is not surprising, since in the first optimization approach, the maximization of *T_dec_* was almost fully accomplished (the partial desirability value was equal to 0.9).

Subsequently, a third optimization run was tested, targeting the maximization of *TS* and a *T_dec_* equal or higher than 300 K, which represents a reasonable increase of the decomposition temperature. Results are presented in Figure S5 of the Supplementary Materials file. The criterion for *T_dec_* was fully met, while a desirability value of 0.89 was achieved for *TS*. Although similar property values with the first run were obtained (*TS* = 758 MPa and *T_dec_* = 300 °C compared with *TS* = 763 MPa and *T_dec_* = 312 °C of the first approach), the predicted composition was rather different (3.85% wt. for the antioxidant content, 1% wt. for the SWCNT content and *λ* equal to 21 compared to 8.0% wt. antioxidant content, 0.77% wt. filler content and drawing ratio equal to 21 from the first approach).

**Figure 10:**
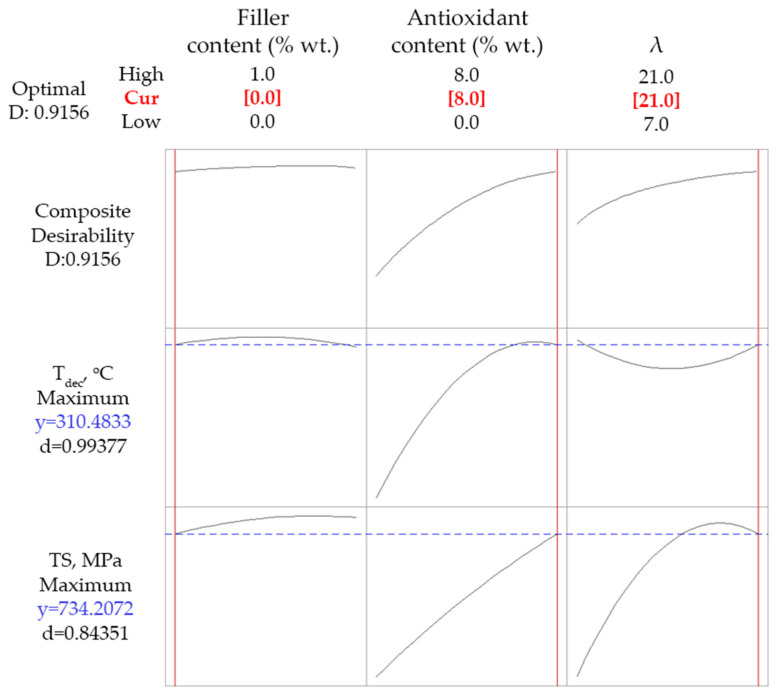
Constrained optimization results for simultaneous maximization of *TS* and *T_dec_*, imposing the absence of SWCNTs.

Considering the increased cost of carbon nanotubes, an interesting question arises: Does the addition of SWCNT significantly improve both investigated properties, i.e., *TS* and *T_dec_*? In order to answer this question, a final optimization run was executed targeting maximization of both *TS* and *T_dec_*, but imposing the absence of carbon nanotubes (thus holding the SWCNT content equal to 0% wt.). The optimization results using this constrain are presented in Figure 10. The model predicted the achievement of *TS* equal to 734 MPa and *T_dec_* equal to 310 K (using no SWCNTs and maximum antioxidant content and drawing ratio). Having in mind the results of Figure 9, the addition of 0.77% wt. of SWCNTs increased *TS* by 29 MPa and *T_dec_* by 1 K. This is a non-negligible improvement for *TS*; however, one should have also in mind that the tensile strength of non-drawn PP fibers ranges between 30–40 MPa [11]. Consequently, it is apparent that drawing has a tremendous effect on mechanical strength, which renders the contribution of fillers rather small.

#### 4.2.4. Verification—Comparison with Literature

As mentioned in Section 4.1.4 for the composites with talc, the Box-Behnken approach indirectly accounts for the repeatability of experimental results through three independent repetitions of a single experiment (see experiments 13, 14, and 15 of Table 5). As shown by the obtained values of Table 5, in all three cases there was an excellent repeatability considering the melting temperature (*T_m_*), while the standard deviation of the three values for the onset decomposition temperature was 2.6 °C and for the tensile strength 43.4 MPa. The latter value may indicate a rather poor dispersion of the filler in the polymer matrix.

In order to verify the model predictions, the model was applied to predict the *TS* of drawn fibers (using a drawing ratio of 14) containing 1% wt. SWCNTs and 4% wt. of the antioxidant masterbatch (0.82% of the active ingredient). Subsequently, a verification experiment was performed. The model predicted a *TS* equal to 632 MPa, while the corresponding experimental value was 750 ± 139 MPa (average of ten measurements from random pieces of fibers ± standard deviation). The high value of standard deviation for the *TS* measurements indicates poor homogeneity of the produced fibers. As already mentioned in Section 4.2.2, this most likely arises from the polar groups of the antioxidant (combination of phenolic and phosphite type) that interact with the filler [11] and, thus, hinder the PP-SWCNT interactions, resulting in poorer dispersion.

From the values presented in Table 5, it can be seen that samples with tensile strength up to 800 MPa were produced. This value is quite high. In the literature, among the highest values for PP drawn fibers that have been reported is a value of 1000 MPa [36]. This was achieved by drawing at a lower temperature (95 °C) than the crystallization temperature, using a drawing ratio of 5 and 0.2% carbon nanotubes content. However, the crystallinity (as indicated by the heat of fusion) of these fibers only slightly increased, or even decreased after drawing. Possibly, such observations [36] are related to the low temperature of drawing. In another study [6], rather low differences were reported between the tensile strength of neat PP (398 MPa) and PP-MWNT (0.5% MWNT) composite drawn fibers (416 MPa) using a drawing ratio of 8. Although PP could be drawn at a drawing ratio of 12, this was not possible for the PP-MWNT composite fibers. Better results were obtained (up to 590 MPa) by using 5% PP-g-MA as compatibilizer and 0.5% amine functionalized MWNT (a-MWNT) [6]. Additionally, similarly to our results, Tambe et al. [6] observed that the tensile strength shows a maximum upon increasing the drawing ratio. Finally, besides any difference in molecular weight and processing, the drawing temperature seemed to be a key factor for the higher values of the current study (806 MPa).

## 5. Conclusions

In this study, optimization of the thermal and mechanical properties of polypropylene composite drawn fibers with two different types of fillers, talc and SWCNTs, was performed based on experiments that were selected through the Box-Behnken method. The drawing ratio, the filler content, and the antioxidant or the compatibilizer content were used as design variables, while tensile strength and decomposition temperature were considered as response variables. In general, quite high values of tensile strength (up to ~800 MPa) were achieved at high drawing ratios (higher than 14).

Considering the composites with talc, it was shown that the simultaneous maximization of mechanical strength and thermal stability could not be achieved due to multiple effects that arise from the interactions between the various additives (talc, compatibilizer, and antioxidant). The addition of talc improved the mechanical strength of fibers drawn at a low ratio (7 in this study), while, at higher drawing ratios, talc particles most likely interfere with the increased chain alignment and, thus, any positive effect on tensile strength by the presence of talc itself cannot compensate for its negative effect on chain alignment.

On the contrary, the optimization targeting maximization of both tensile strength and thermal stability was feasible in the case of SWCNT composite fibers. It was found that the addition of carbon nanotubes improved the tensile strength; however, such an improvement was rather small compared with the tremendous increase of tensile strength due to drawing. However, there is an upper limit in the positive effect of drawing. This upper limit is expected to vary with the characteristics of various common industrial additives such as the antioxidant.

Overall, the results of this study, as well as the results of the previous studies of this series [11,12], imply that additives, such as compatibilizers, antioxidants, UV protection, or coloring agents, which are often used in industrial practice and which are not considered in most research studies, may have a strong effect, often positive, on the properties of drawn polymer fibers.

## Figures and Tables

**Table 1:** Important characteristics of the used materials.

Material	Abbreviation	Characteristics ^1,2,3,4^	Supplier
Isotactic PP (ECOLEN HZ42Q)	PP	*MFI* = 18 g/10 min, *TS* = 33 MPa, *T_m_* = 168–171 °C	Hellenic Petroleum S.A., Thessaloniki, Greece
Masterbatch with compatibilizer (Bondyram 1001)	MA	PP grafted with maleic anhydride (PP-g-MA). MA content 1%, *MFI* = 100 g/10 min, *T_m_* = 160 °C	Polyram Plastic Industries LTD, Gilboa, Israel
Masterbatch with antioxidant (KRITILEN^®^ AO PP9216)	AO	PP with 20.5% wt. antioxidant (combination of phosphite and phenolic types)	Plastika Kritis S.A., Heraklion, Greece
Masterbatch with ultra-fine talc (KRITILEN^®^ D05-00046)	UT	PP with 30% wt. ultra-fine (*D*_50_ = 0.7 μm) *MFI* ^5^ = 25 g/10 min	Plastika Kritis S.A., Heraklion, Greece
Masterbatch with SWCNT (KRITILEN^®^ D05-00067)	SWCNT	PP with 5% wt. SWCNT *MFI* ^5^ = 25 g/10 min	Plastika Kritis S.A., Heraklion, Greece

^1^ *MFI*: melt flow index, ^2^ *TS*: tensile strength, ^3^ *T_m_*: melting point, ^4^ *D*_50_: Mass-median-diameter, ^5^ MFI of the PP used for the preparation of the masterbatch and not the MFI of masterbatch itself.

**Table 2:** Compositions and drawing ratios for the 15 experiments of the first DoE (composites with talc).

ID	Talc Content (% wt.)	Compatibilizer Content ^a^ (% wt.)	Drawing Ratio, *λ*
1	0	0	8
2	4	0	8
3	0	7.5	8
4	4	7.5	8
5	0	3.75	7
6	4	3.75	7
7	0	3.75	9
8	4	3.75	9
9	2	0	7
10	2	7.5	7
11	2	0	9
12	2	7.5	9
13	2	3.75	8
14	2	3.75	8
15	2	3.75	8

^a^ On masterbatch base.

**Table 3:** Compositions and drawing ratios for the 15 experiments of the second DoE (composites with SWCNTs).

ID	SWCNT Content (% wt.)	Antioxidant Content ^a^ (% wt.)	Drawing Ratio, *λ*
1	0	0	14
2	1	0	14
3	0	8	14
4	1	8	14
5	0	4	7
6	1	4	7
7	0	4	21
8	1	4	21
9	0.5	0	7
10	0.5	8	7
11	0.5	0	21
12	0.5	8	21
13	0.5	4	14
14	0.5	4	14
15	0.5	4	14

^a^ On masterbatch base.

**Table 4:** DoE1 results (composites with talc).

ID	Talc Content (% wt.)	Compatibilizer Content ^a^ (% wt.)	Drawing Ratio, *λ*	*TS* (ΜΡa)	*T_dec_* (°C)	Δ*H* (J g^−1^)	*T_m_* (°C)
1	0	0.00	8	451 ± 56	303	104.8	163
2	4	0.00	8	419 ± 45	316	101.6	164
3	0	7.50	8	481 ± 67	308	100.1	163
4	4	7.50	8	425 ± 69	301	103.5	165
5	0	3.75	7	414 ± 90	291	96.5	163
6	4	3.75	7	376 ± 44	291	96.2	164
7	0	3.75	9	744 ± 43	298	107.2	166
8	4	3.75	9	438 ± 68	304	98.1	161
9	2	0.00	7	363 ± 26	311	99.3	163
10	2	7.50	7	272 ± 79	305	99.7	163
11	2	0.00	9	454 ± 30	301	98.9	163
12	2	7.50	9	439 ± 57	302	98.6	167
13	2	3.75	8	442 ± 37	301	101.9	165
14	2	3.75	8	478 ± 145	297	100.6	165
15	2	3.75	8	433 ± 42	301	100.2	165

^a^ On mastebatch basis.

**Table 5:** DoE2 results (composites with SWCNTs).

ID	SWCNT Content (% wt.)	Antioxidant Content ^a^ (% wt.)	Drawing Ratio, *λ*	*TS* (ΜΡa)	*T_dec_* (°C)	Δ*H* (J g^−1^)	*T_m_* (°C)
1	0	0	14	521 ± 51	268	98.2	171
2	1	0	14	536 ± 77	260	106.0	169
3	0	8	14	806 ± 70	308	107.3	171
4	1	8	14	623 ± 89	305	107.9	169
5	0	4	7	402 ± 21	308	85.1	167
6	1	4	7	345 ± 11	308	86.6	167
7	0	4	21	598 ± 169	302	111.3	169
8	1	4	21	802 ± 227	302	94.3	166
9	0.5	0	7	406 ± 37	283	86.6	165
10	0.5	8	7	357 ± 36	311	84.2	166
11	0.5	0	21	673 ± 74	273	97.9	168
12	0.5	8	21	706 ± 56	310	107.1	167
13	0.5	4	14	596 ± 29	299	91.1	169
14	0.5	4	14	643 ± 45	298	100.5	170
15	0.5	4	14	702 ± 88	304	100.9	170

^a^ On masterbatch basis.

## Data Availability

Not applicable.

## Supplementary Materials

The following are available online at https://www.mdpi.com/article/10.3390/polym14071329/s1, Figure S1: Representative stress-strain curves for SWCNT and talc composites line), Figure S2: Representative DSC plots for SWCNT and talc composites, Figure S3: Representative TGA plots for SWCNT and talc composites, Figure S4: Optimization results targeting maximum tensile strength, *TS*, for composites with SWCNTs, Figure S5: Optimization results targeting maximum tensile strength, *TS*, and onset decomposition temperature, *T_dec_*, at least equal to 300 °C for composites with SWCNTs, Table S1: *R-sq* and *p* values for the fitting model for the case of PP-talc, Table S2: *R-sq* and *p* values for the fitting model for the case of PP-CNT.
