# Supplementary material for: Surface Response Analysis for the Optimization of Mechanical and Thermal Properties of Polypropylene Composite Drawn Fibers with Talc and Carbon Nanotubes
Source: Polymers (Basel). 2022 Mar 25;14(7):1329. doi: 10.3390/polym14071329 (PMC9002490; doi:10.3390/polym14071329)
Supplement: Supplementary file 1 [file polymers-14-01329-s001.zip › polymers-1638184-Supplementary.pdf]

polymers MDPI

Supplementary Information for

Surface Response Analysis for the Optimization of Mechanical and Thermal Properties of Polypropylene Composite Drawn Fibers with Talc and Carbon Nanotubes

Konstantinos Leontiadis [1], Costas Tsioptsias [1,*], Stavros Messaritakis [2], Aikaterini Terzaki [2], Panagiotis Xidas [3], Kyriakos Mystikos [3], Evangelos Tzimpilis [1] and Ioannis Tsivintzelis [1,*]

[1] Department of Chemical Engineering, Aristotle University of Thessaloniki, University Campus, GR-54124 Thessaloniki, Central Macedonia, Greece; leontiad@cheng.auth.gr (K.L.); tzimpi@auth.gr (E.T.)
[2] Plastika Kritis S.A., R Street, Industrial Area of Heraklion, GR-71408 Heraklion, Crete, Greece; messaritakis@plastikakritis.com (S.M.); terzaki@plastikakritis.com (A.T.)
[3] Thrace Nonwovens & Geosynthetics S.A., Magiko, GR-67100 Xanthi, Western Thrace, Greece; pxidas@thraceplastics.gr (P.X.); kmystikos@thraceplastics.gr (K.M.)
* Correspondence: ktsiopts@gmail.com (C.T.); tioannis@cheng.auth.gr (I.T.)

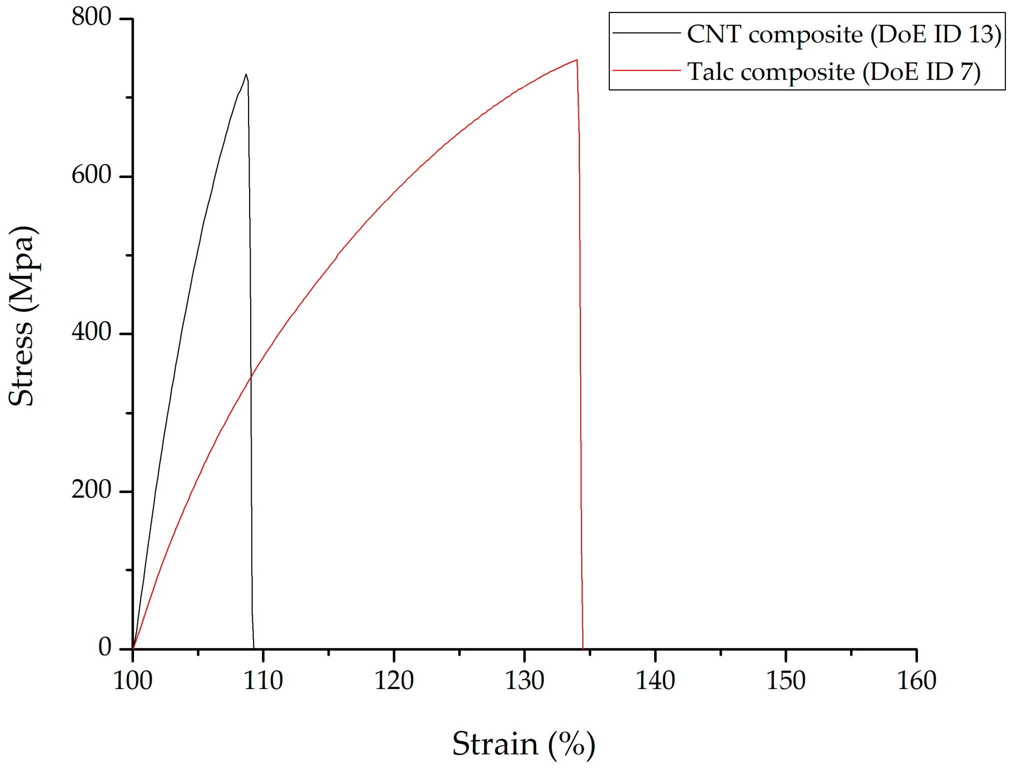

Figure S1. Representative stress-strain curves for SWCNT composites (black line) and talc composites (red line).

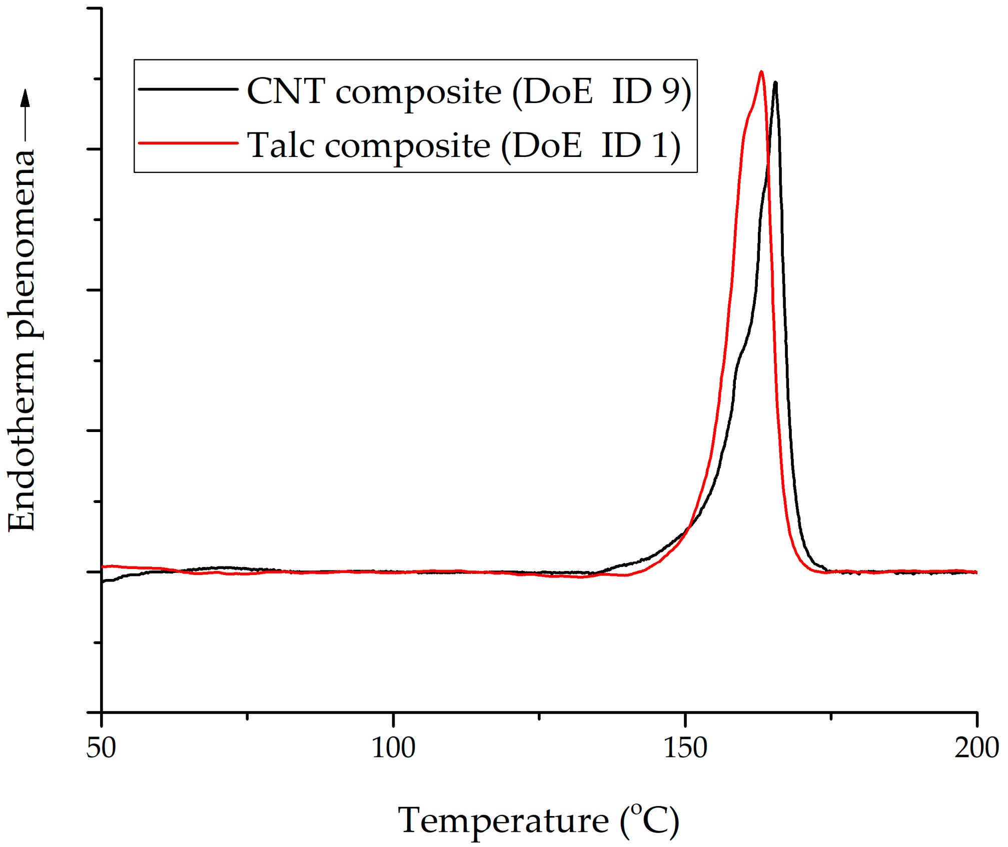

Figure S2. Representative DSC plots for SWCNT composites (black line) and talc composites (red line).

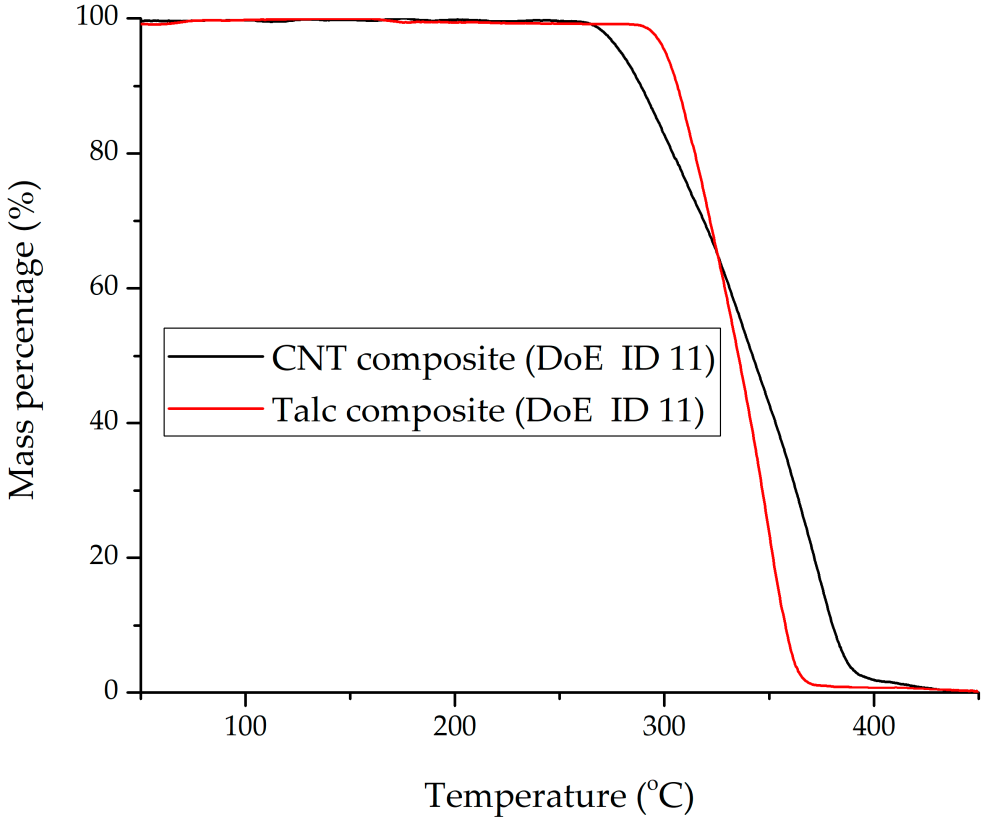

Figure S3. Representative TGA plots for SWCNT composites (black line) and talc composites (red line).

Table S1. *R-sq* values and *P* values for the fitting model for the case of PP-talc.

	TS	T_{dec}	ΔH	T_m
R-sq	0.897	0.7628	0.6827	0.751
P-value	0.049	0.271	0.444	0.296

Table S2. *R-sq* values and *P* values for the fitting model for the case of PP-CNT.

	TS	T_{dec}	ΔH	T_m
R-sq	0.8832	0.9778	0.8874	0.9617
P-value	0.064	0.001	0.059	0.005

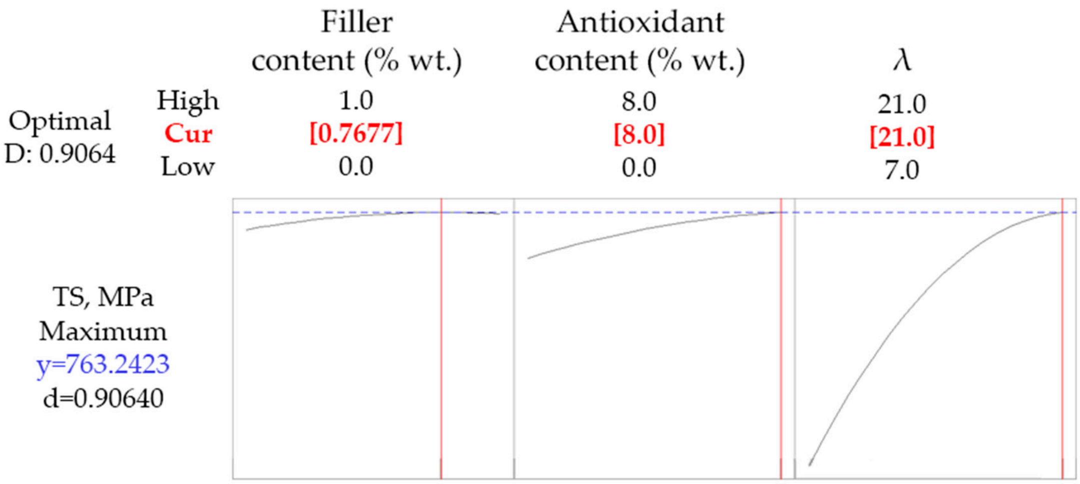

Figure S4. Optimization results targeting maximum tensile strength, *TS*, for composites with SWCNTs.

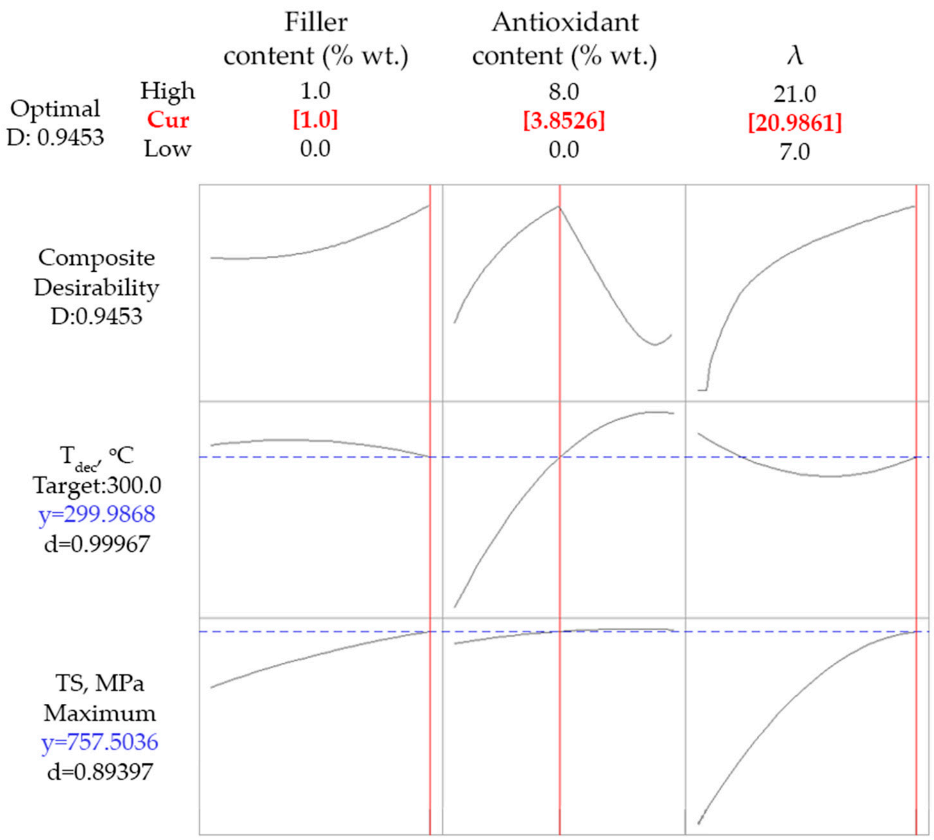

Figure S5. Optimization results targeting maximum tensile strength, *TS*, and onset decomposition temperature, T_{dec}, at least equal to 300 °C for composites with SWCNTs.
